# Feasibility of biodiesel production and CO_2_ emission reduction by *Monoraphidium dybowskii* LB50 under semi-continuous culture with open raceway ponds in the desert area

**DOI:** 10.1186/s13068-018-1068-1

**Published:** 2018-04-02

**Authors:** Haijian Yang, Qiaoning He, Chunxiang Hu

**Affiliations:** 1 Key Laboratory of Algal Biology, Institute of Hydrobiology, Chinese Academy of Sciences (CAS), Wuhan, 430072 China; 2 University of Chinese Academy of Sciences, Beijing, 100039 China

**Keywords:** Microalgae, Lipid production, Semi-continuous culture, CO_2_ fixation, Open raceway ponds, Desert area, Evaporation

## Abstract

**Background:**

Compared with other general energy crops, microalgae are more compatible with desert conditions. In addition, microalgae cultivated in desert regions can be used to develop biodiesel. Therefore, screening oil-rich microalgae, and researching the algae growth, CO_2_ fixation and oil yield in desert areas not only effectively utilize the idle desertification lands and other resources, but also reduce CO_2_ emission.

**Results:**

*Monoraphidium dybowskii* LB50 can be efficiently cultured in the desert area using light resources, and lipid yield can be effectively improved using two-stage induction and semi-continuous culture modes in open raceway ponds (ORPs). Lipid content (LC) and lipid productivity (LP) were increased by 20% under two-stage industrial salt induction, whereas biomass productivity (BP) increased by 80% to enhance LP under semi-continuous mode in 5 m^2^ ORPs. After 3 years of operation, *M. dybowskii* LB50 was successfully and stably cultivated under semi-continuous mode for a month during five cycles of repeated culture in a 200 m^2^ ORP in the desert area. This culture mode reduced the supply of the original species. The BP and CO_2_ fixation rate were maintained at 18 and 33 g m^−2^ day^−1^, respectively. Moreover, LC decreased only during the fifth cycle of repeated culture. Evaporation occurred at 0.9–1.8 L m^−2^ day^−1^, which corresponded to 6.5–13% of evaporation loss rate. Semi-continuous and two-stage salt induction culture modes can reduce energy consumption and increase energy balance through the energy consumption analysis of life cycle.

**Conclusion:**

This study demonstrates the feasibility of combining biodiesel production and CO_2_ fixation using microalgae grown as feedstock under culture modes with ORPs by using the resources in the desert area. The understanding of evaporation loss and the sustainability of semi-continuous culture render this approach practically viable. The novel strategy may be a promising alternative to existing technology for CO_2_ emission reduction and biofuel production.

**Electronic supplementary material:**

The online version of this article (10.1186/s13068-018-1068-1) contains supplementary material, which is available to authorized users.

## Background

Renewable and environmentally friendly alternative fuels are urgently needed for future industrial development, because of the diminishing world oil reserves and the environmental deterioration associated with fossil fuel consumption [1, 2]. Microalgae are increasingly considered as feedstock for next-generation biofuel production because of their many excellent characteristics, such as broad environmental adaptability, short growth period, high photosynthetic efficiency, and high-quality lipid [3, 4]. However, the commercial feasibility of microalgal biodiesel is limited because only few microalgal strains can be grown reliably with high lipid content (LC) outdoors. Lipid productivity (LP) under outdoor conditions is significantly lower than that in the laboratory due to pollution from other microorganisms and fluctuations in environmental parameters [5–7]. Large-scale outdoor cultivation using sunlight is the only solution for the sustainable industrial production of microalgal biofuel [8]. Therefore, an essential prerequisite to achieve the industrial-scale application of microalgal biofuel is the selection of robust and highly productive microalgal strains with relatively high LC outdoors.

Two cultivation systems are commonly used for large-scale outdoor microalgal cultivation: the open system (e.g., open raceway ponds, ORPs) and the closed photobioreactor system (e.g., tubular, flat plate or column photobioreactors) [1, 9–11]. Compared with closed photobioreactors, ORPs consume less energy and require lower investment and production costs for microalgal cultivation [12]. Although microalgal cultivation in ORPs offers many advantages, the high cost of cultivation systems impedes the commercialization for lipid production. Thus, developing an economically feasible culture mode to increase the lipid production and thereby reduce cultivation costs is necessary [7, 13].

Increasing LP via culture modes can reduce the costs and enhance the economic feasibility of microalgal biodiesel production. The photoautotrophic two-stage cultivation mode is a highly promising approach to increase lipid production in photobioreactors by improving LCs [14–16]. However, only few studies have employed this mode in ORPs [17]. The semi-continuous mode is a simple and efficient strategy to increase lipid production in microalgal biomass by continuously increasing biomass [7, 18]. This mode can avoid a low cell division rate at the early exponential stage and light limitation at the late stationary stage. Furthermore, it maintains the microalgal culture under exponential growth conditions, resulting in enhanced biodiesel production [7]. However, the number of cycles for semi-continuous culture is limited because of the different nutrient consumption rates of algal cells. Thus, exploring the adequate cultivation time of microalgal cells under whole semi-continuous cultivation mode is important to evaluate their survivability.

The large-scale cultivation of microalgae requires large areas of land and water resources. Arid and semiarid regions account for 41% of the global land area [19]. Thus, cultivating microalgae in desertification areas avoids competition with food crops for arable land and water. In addition, the unique climatic conditions (strong solar radiation, long sunshine duration, and large day and night temperature difference) in deserts are beneficial to the accumulation of dry weight (DW) in the cells. Compared with other crops, microalgae are more compatible with desert conditions. Furthermore, cyanobacteria and green microalgae can be stably and efficiently cultivated in desert areas [13, 20, 21]. Therefore, microalgal cultivation is an effective means to utilize desert lands and sunshine.

In the current work, the ability of three microalgae to produce high lipid indoors was determined in a 5 m^2^ (1000 L) ORP to select for high environmental adaptability and lipid accumulation capability in the desert area. The influences of two-stage cultivation mode and semi-continuous mode on cell growth, CO_2_ fixation rate, and evaporation rate were first investigated under 5 m^2^ (1000 L) ORP. Algal strain growth was scaled up to a 200 m^2^ (40,000 L) ORP with semi-continuous mode to determine cycle times. Outdoor cultivation test at different times was conducted to assess the stability of the algal strain in long-term semi-continuous operations. Finally, the energy consumption of life cycle was analyzed to assess the feasibility of biodiesel production and CO_2_ mitigation in desert area.

## Methods

### Organism

*Monoraphidium dybowskii* LB50 and *Micractinium* sp. XJ-2 were provided by Prof. Xudong Xu of the Institute of Hydrobiology, the Chinese Academy of Sciences. *Podohedriella falcata* XJ-176 was isolated from Xinjiang Taxi River Reservoir (Additional file 1: Figure S1). The stock cultures were maintained indoors in a sterilized BG11 medium containing 1.5 g NaNO_3_, 40 mg K_2_HPO_4_, 75 mg MgSO_4_·7H_2_O, 20 mg Na_2_CO_3_, 36 mg CaCl_2_·2H_2_O, 6 mg ammonium ferric citrate, 6 mg ammonium citrate monohydrate, 1 mg EDTA, 2.86 μg H_3_BO_3_, 1.81 μg MnCl_2_·4H_2_O, 0.222 μg ZnSO_4_·7H_2_O, 0.39 μg Na_2_MoO_4_·2H_2_O, 0.079 μg CuSO_4_·5H_2_O, and 0.050 μg CoCl_2_·6H_2_O in 1 L water.

### Experimental setup

All experiments were conducted in the Dalate Banner of Inner Mongolia Autonomous Region at the East edge of Hobq Desert (40°22′23.4″N 109°50′57.7″E) for 3 years (Additional file 1: Figure S1). Two scales of ORPs at 5 and 200 m^2^ were utilized. The length, width, and maximum depth were 4.80, 1.05, and 0.60 m and 34.50, 5.80, and 0.60 m in 5 and 200 m^2^ illuminated areas of ORP, respectively (Additional file 1: Figure S1). The culture depth in raceway ponds was set to 20 cm, with 1000 and 40,000 L culture volumes. A stainless steel paddlewheel, 0.80 m in diameter, was used for the circulation of the cultures in 5 and 200 m^2^ ORPs at 0.35 and 0.25 m s^−1^, respectively. Microalgae were cultivated using a modified BG11 medium containing 0.25 g L^−1^ urea, but 0.1 M NaHCO_3_ was added to the medium used for *M. dybowskii* LB50. The medium was thoroughly compounded with groundwater. A series of scale-up pre-cultivation was employed (Additional file 1: Figure S1). Water in the system was replenished every day to prevent serious evaporative losses in the open raceway system. Cell concentration measured as an OD_680_ of 0.1 was inoculated into the culture in 5 and 200 m^2^ ORPs.

After pre-cultivation, the batch culture was conducted with three microalgae in 5 m^2^ ORP (1000 L) to select the optimal stain for lipid production.

For two-stage salt induction culture in 5 m^2^ ORPs, *M. dybowskii* LB50 was cultivated in 5 m^2^ ORPs outdoors. On the 10th day, which is at the late-exponential growth phase, NaCl and industrial salts (Hubei Guangyan Lantioan salt chemical co., Ltd, China. Additional file 2: Table S1) were added at final concentrations of 0 and 20 g L^−1^. Industrial salts, often referred in China to NaCl, NaOH (caustic soda), and Na_2_CO_3_ are widely used in the industry. In the current study, the main component of industrial salt was NaCl. Industrial salt can be inexpensive and is easily produced because of the low purity. Day 0 was assumed as the time of salt addition.

For semi-continuous cultivation, further experiments were conducted with semi-continuous mode in two ORP scales. Two-thirds of the culture was harvested, and the remaining culture was used as the seed for subsequent batches and replaced by the same volume of nutrition-rich growth media containing half of the urea concentration. The algal culture was harvested every 3 or 4 days. The semi-continuous experiment was carried out in a 200 m^2^ ORP for a month.

The water used for algal cultivation was pumped from the ground and contained 89.39 ppm Na^+^, 62.92 ppm SO_4_^2+^, and low levels of K^+^ (1.69 ppm), Mg^2+^(13.65 ppm), Ca^2+^ (12.66 ppm), Cl^−^ (24.12 ppm), and NO_3_^−^ (1.41 ppm) [13].

### Analytical procedures

#### Biomass measurement

Biomass productivity (BP, mg L^−1^ day^−1^) was calculated according to Eq. (1):

1 $${\text{BP}} = \, \left( {B2\,{-}\,B1} \right)/ \, t,$$

where *B*2 and *B*1 represent the DW biomass density at time *t* (days) and at the start of the experiment, respectively.

Algal density was determined by measuring the OD_680_—the optical density of algae at 680 nm. The relationships between the DW (g L^−1^) and the OD_680_ values of the algae were described using Eqs. (2–4):

2 $${\text{DW}} = 0.2122 \times {\text{OD}}\,-\,0.0037\;\;\;\;R^{2} = 0.9888\;\;\;\;\left( {M. \, dybowskii\;\;\;{\text{LB50}}} \right),$$

3 $${\text{DW}} = 0.2866 \times {\text{OD}}\,-\,0.0052\;\;\;\;R^{2} = 0.991\;\;\;\left( {Micractinium\;{\text{sp}}.{\text{ XJ-}}2} \right),$$

4 $${\text{DW}} = 0.217 \times {\text{OD}}\,-\,0.0089\;\;\;\;R^{2} = 0.9947\;\;\;\left( {P. \, falcate\;\;{\text{XJ-}}176} \right).$$

The cells were harvested by centrifugation and baked in an oven.

#### Lipid analysis

Total lipid was extracted from approximately 80–100 mg of the dried algae (*w*_1_) using a Soxhlet apparatus, with chloroform–methanol (1:2, v/v) as the solvent. Total lipid was transferred into a pre-weighed beaker (*w*_2_) and blow-dried in a fume cupboard. The lipid was dried to a constant weight in an oven at 10 °C and weighed (*w*_3_).

LC (%) and the LP (mg L^−1^ day^−1^) were determined according to Eqs. (5, 6):

5 $${\text{LC }}\left( \% \right) \, = \, (w_{3} \,{-}\,w_{2} )/w_{1} \times 100,$$

6 $${\text{LP }}\left( {{\text{mg}}\;{\text{L}}^{ - 1} \;{\text{day}}^{ - 1} } \right) \, = {\text{ BP}} \times {\text{LC}}.$$

### Determination of urea concentration

Urea concentration was determined following the protocol outlined by Beale and Croft [22]. The liquid sample collected from the raceway pond was filtered using a 0.22 μm-pore filter and then diluted 60-fold with deionized water for each sample. The sample was collected and mixed with 1 volume of diacetylmonoxime–phenylanthranilic acid reagent (1 volume of 1% w/v diacetylmonoxime in 0.02% acetic acid and 1 volume of phenylanthranilic acid in 20% v/v ethanol with 120 mM NaCO_3_). Exactly, 1 mL of activated acid phosphate (1.3 M NaH_2_PO_4_, 10 mM MnCl_2_, 0.4 mM NaNO_3_, 0.2 M HCl in 31% v/v H_2_SO_4_) was added before incubation in boiling water for 15 min. The tubes were left to cool, and their OD_520_ were determined using a UV/Vis spectrophotometer.

### Determination of pH, irradiance, conductivity, and evaporation

The temperature, conductivity and pH of the culture medium were determined daily by utilizing respective sampling probes (YSI Instruments, Yellow Springs, Ohio, USA). Irradiance was measured with a luxmeter (Hansatech Instruments, Norfolk, UK).

The depth at four fixed positions was determined in the raceway ponds every day, and evaporation (L m^−2^ day^−1^) was calculated according to Eq. (7):

7 $${\text{Evaporation}} = (h2\,{-}\,h1) \times S/\left( {t \times S} \right),$$

where *h*2 and *h*1 represent the average depth at time *t* (days) and at the start of the experiment, respectively. *S* represents the area of the raceway ponds.

### Determination of CO_2_ fixation rate

According to the mass balance of microalgae, the fixation rate of CO_2_ (mg L^−1^ day^−1^, g m^−2^ day^−1^) was calculated from the relationship between the carbon content and volumetric growth rate of the microalgal cell, as indicated in Eq. (8):

8 $${\text{CO}}_{2} \;{\text{fixation}}\;{\text{rate }} = {\text{ BP}} \times C_{\text{carbon}} \times \left( {M_{{{\text{CO}}_{ 2} }} /M_{\text{c}} } \right),$$

where BP is in mg L^−1^ day^−1^ or g m^−2^ day^−1^; *C*_carbon_ is the carbon content of the biomass (g g^−1^), as determined by an elemental analyzer (Elementar Vario EL cube); $M_{{{\text{CO}}_{ 2} }}$ is the molar mass of CO_2_; and *M*_C_ is the molar mass of carbon (Additional file 3: Table S2).

### Net energy ratio (NER) and energy balances

NER is defined as the ratio of the energy produced over primary energy input as represented in Eq. (9):

9 $${\text{NER}} = \sum {\text{Energy produced }}\left( {\text{lipid or biomass}} \right)/\sum {\text{Energy requirements}} .$$

On the basis of the data obtained in the 200 m^2^ ORP for cultivating *M. dybowskii* LB50 for 1 year, NER is estimated using the method discussed by Jorquera et al. [23].

Energy balance is defined as the difference between energy produced and primary energy input, as represented Eq. (10):

10 $${\text{Energy balance}} = \sum {\text{Energy produced }}\left( {\text{lipid or biomass}} \right){-}\sum {\text{Energy requirements}} .$$

### Statistical analysis

The values were expressed as mean ± standard deviation. The data were analyzed by one-way ANOVA using SPSS (version 19.0). Statistically significant difference was considered at *p *< 0.05.

## Results and discussion

### Growth, lipid accumulation, and CO_2_ fixation rate of the three microalgae in 5 m^2^ ORPs outdoors

**Fig. 1:**
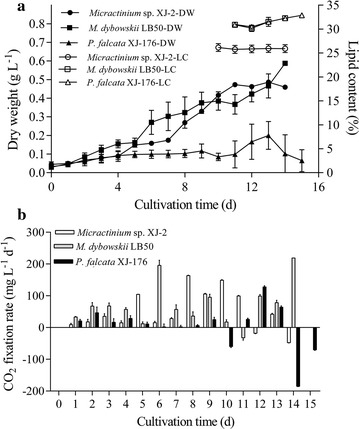
Biomass, lipid content (**a**), and CO_2_ fixation rate (**b**) of *M. dybowskii* LB50, *Micractinium* sp. XJ-2, and *P. falcata* XJ-176 in 5 m^2^ ORPs. *DW* dry weight, *LC* lipid content

Three strains of potential microalgae (Additional file 4: Table S3) were grown in 5 m^2^ ORPS to evaluate their lipid accumulation and CO_2_ fixation potential. As shown in Fig. 1, the BPs of *M. dybowskii* LB50 and *Micractinium* sp. XJ-2 were both 42 mg L^−1^ day^−1^ (8 g m^−2^ day^−1^), whereas *P. falcata* XJ-176 cannot be reliably cultured outdoors. The LC of *M. dybowskii* LB50 (30%) was higher than that of *Micractinium* sp. XJ-2 (*p* < 0.05). Thus, the LP of *M. dybowskii* LB50 (2.6 g m^−2^ day^−1^) was also higher than that of *Micractinium* sp. XJ-2 (2.28 g m^−2^ day^−1^). The CO_2_ fixation rates of *M. dybowskii* LB50, *Micractinium* sp. XJ-2, and *P. falcata* XJ-176 were 59, 41, and 19 mg L^−1^ day^−1^ (12, 8, and 4 g m^−2^ day^−1^, Additional file 5: Table S4), respectively. During the time course of culture, CO_2_ fixation rate was low at the beginning and stable stage and was the highest at the exponential growth stage, reaching 163 mg L^−1^ day^−1^. At the late growth stage, CO_2_ fixation rate was negative, indicating that the microalgal cells did not grow or died, releasing large amounts of CO_2_ possibly through respiratory metabolism.

Microbial contamination during large-scale algal cultivation can significantly and consistently reduce biomass production. In this context, eukaryotic contaminants, such as amoebae, ciliates, and rotifers, and clusters of cells based on microscopy were found to cause biomass deterioration in *P. falcata* XJ-176 cultivation. In the current study, this phenomenon was rarely observed during the cultivation of *M. dybowskii* LB50 and *Micractinium* sp. XJ-2. These results showed that the two species demonstrate high environmental tolerance, especially to the high light intensity in the desert (Additional file 6: Figure S2), and could inhibit the excessive growth of bacteria [16, 24]. Consequently, *M. dybowskii* LB50 exhibited improved lipid accumulation potential outdoors, particularly during cultivation in the desert.

### Two-stage induction culture of microalgae

In addition to selecting a fast-growing strain with high LC, improving the LC or biomass to increase lipid yield is also necessary to enhance the economic feasibility of microalgae-based CO_2_ removal and biodiesel production [13, 16, 25]. LC can be improved through many ways [7, 26], among which two-stage salt induction is very effective [27]. In our previous study, the LC of *M. dybowskii* LB50 was increased by 10% through NaCl induction in 140 L photobioreactors outdoors [16]. However, few studies on NaCl induction in ORPs have been conducted [17].

**Fig. 2:**
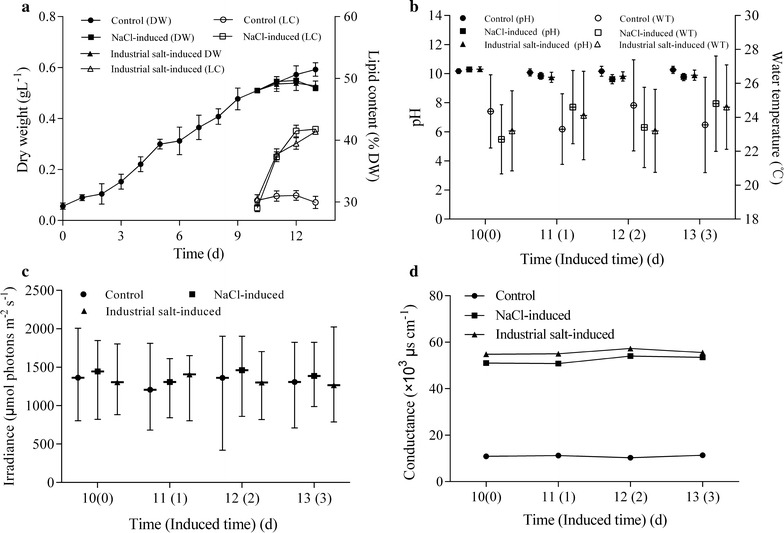
*Monoraphidium dybowskii* LB50 cultivated via NaCl and industrial salt induction in 5 m^2^ ORPs. Biomass and lipid content (**a**), pH and water temperature (**b**), irradiance (**c**), and conductance (**d**)

Figure 2 shows that the biomass was not significantly decreased on the first day of NaCl and industrial salt induction (*p* > 0.05), but was significantly reduced on the third day (*p* < 0.05). The effect of industrial salt induction on LC was similar to that of NaCl. LC increased by 7% on day 1 of induction and by 10% on day 2 of induction. Thus, LP was 3.3 g m^−2^ day^−1^ without significant difference within 1 or 2 days of induction. Only 1 day was required for induction to shorten the culture period. Meanwhile, CO_2_ fixation rate was 78 mg L^−1^ day^−1^ at the time course of induction (Table 1). The pH of the culture liquid did not significantly change, after adding NaCl or industrial salt, but the conductivity increased by five times after adding salt ions (Additional file 2: Table S1). Consequently, the two-stage industrial salt induction culture mode in ORPs favorably increased the LC and reduced the costs.

**Table 1:** LC, VBP, ABP, VLP, ALP, and CO_2_ fixation rate of *M. dybowskii* LB50 under two-stage induction culture in 5 m^2^ ORPs

	0–11 days			0–12 days		
	Control	NaCl induced	Industrial salt induced	Control	NaCl induced	Inustrial salt induced
LC (%)	30.95 ± 0.83	37.31 ± 0.79	37.70 ± 0.89	31.05 ± 0.79	41.48 ± 0.95	39.46 ± 0.94
VBP (mg L^−1^ day^−1^)	44.02 ± 1.90	44.46 ± 2.96	43.55 ± 2.78	43.00 ± 0.54	41.04 ± 1.87	40.20 ± 0.02
ABP (g m^−2^ day^−1^)	8.80 ± 0.38	8.89 ± 0.79	8.71 ± 0.56	8.60 ± 0.11	8.21 ± 0.37	8.04 ± 0.09
VLP (mg L^−1^ day^−1^)	13.62 ± 0.16	16.59 ± 0.23	16.42 ± 0.25	13.35 ± 0.04	17.02 ± 0.18	15.86 ± 0.25
ALP (g m^−2^ day^−1^)	2.72 ± 0.31	3.32 ± 0.06	3.28 ± 0.49	2.67 ± 0.01	3.41 ± 0.06	3.17 ± 0.01
CO_2_ fixation rate (mg L^−1^ day^−1^)	78.94 ± 3.40	79.74 ± 5.31	78.11 ± 4.98	77.11 ± 0.96	73.59 ± 3.36	72.11 ± 1.21
CO_2_ fixation rate (g m^−2^ day^−1^)	15.79 ± 0.68	15.95 ± 1.06	15.62 ± 1.00	15.42 ± 0.19	14.72 ± 0.67	14.42 ± 0.09

*VBP* volume biomass productivity, *ABP* areal biomass productivity, *VLP* volume lipid productivity, *ALP* areal lipid productivity, *LC* lipid content

Two-stage cultivation has been performed in closed photobioreactors outdoors. *Tetraselmis* sp. and *Chlorella* sp. were cultured in 120 L closed photobioreactors, and lipid productivities of microalgae were increased by suitable CO_2_ concentration [11, 28]. Moreover, NaCl induction in the column photobioreactors was favorable [16]. However, these reports have not been verified in ORPs. Kelley [29] reported LC can be increased by using a two-step method involving N deficiency and light conversion in 3 m^2^ ORPs. LP can also be increased by NaCl induction during dual mode cultivation of mixotrophic microalga in culture tubes [17]. In this study, we confirmed that LC was significantly increased not only in the open runway pool (1000 L), but also with industrial salt induction.

### Semi-continuous culture of microalgae

#### Semi-continuous culture in 5 m^2^ ORPs

Given its convenient operation and cost-effectiveness, semi-continuous cultivation is also a good choice [30]. Semi-continuous cultivation has attracted considerable attention in energy microalgae [7, 18, 31]. Unfortunately, the culture medium used in semi-continuous cultivation cannot be reused for an unlimited number of times because of the difference in nutrients consumption rate of cells. Portions of the nutrient concentration excessively increase with culture time and eventually inhibit cell growth.

**Fig. 3:**
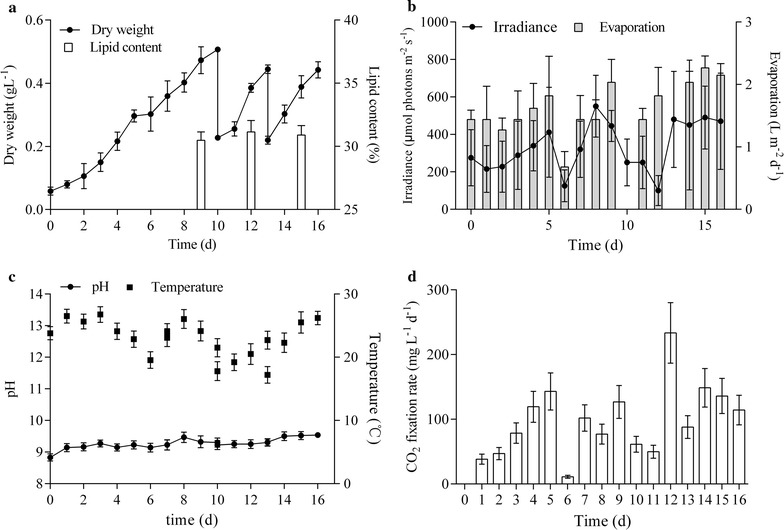
Biomass and lipid content (**a**), irradiance and evaporation (**b**), pH and water temperature (**c**), and CO_2_ fixation rate (**d**) of *M. dybowskii* LB50 under semi-continuous mode in the 5 m^2^ ORP. *DW* dry weight, *LC* lipid content

In the 1000 L ORP, the BP increased from 44.86 to 74.16 mg L^−1^ day^−1^ after repeated culture, and the LC remained stable at 30% in *M. dybowskii* LB50 (Fig. 3). Finally, areal LP (ALP) increased from 2.73 to 4.58 g m^−2^ day^−1^ (Table 2), and the CO_2_ fixation rate increased from 16.1 to 26.7 g m^−2^ day^−1^ after repeated culture. During the whole semi-continuous culture, the CO_2_ fixation rate reached 23 g m^−2^ day^−1^ (114 mg L^−1^ day^−1^). The pH of the culture medium did not significantly change (9.14–9.52, Fig. 3c), indicating that the growth consistently improved throughout the semi-continuous culture. However, the fluctuations in light intensity and temperature were large. Increased illumination and prolonged periods of light exposure were favorable factors for microalgal culture in desert areas, but high evaporation due to increased illumination was unfavorable. Evaporation occurred at 1.62 L m^−2^ day^−1^ (Fig. 3). The minimum amount of evaporation was 0.68 L m^−2^ day^−1^ at low temperature and light intensity (day 6, rainy day), whereas the highest evaporation rate was 2.26 L m^−2^ day^−1^ at high temperature and light intensity in the 5 m^2^ ORP.

**Table 2:** LC, VBP, ABP, VLP, ALP, and CO_2_ fixation rate of *M. dybowskii* LB50 under semi-continuous mode in 5 m^2^ ORPs

	0–10 days	11–13 days	14–16 days	0–16 days
LC (%)	30.48 ± 0.67	31.15 ± 0.89	30.9 ± 0.74	30.84 ± 0.34
VBP (mg L^−1^ day^−1^)	44.86 ± 2.1	72.32 ± 3.51	74.16 ± 2.25	63.78 ± 3.05
ABP (g m^−2^ day^−1^)	8.97 ± 0.23	14.46 ± 0.19	14.83 ± 0.26	12.75 ± 0.23
VLP (mg L^−1^ day^−1^)	13.67 ± 0.64	22.53 ± 1.09	22.87 ± 0.7	19.66 ± 0.94
ALP (g m^−2^ day^−1^)	2.73 ± 0.07	4.51 ± 0.06	4.58 ± 0.08	3.93 ± 0.07
CO_2_ fixation rate (mg L^−1^ day^−1^)	80.54 ± 3.77	129.87 ± 6.29	133.65 ± 4.04	114.47 ± 5.47
CO_2_ fixation rate (g m^−2^ day^−1^)	16.09 ± 0.41	24.75 ± 0.34	26.73 ± 0.47	23.1 ± 0.41

The two-stage induction culture exhibited slightly higher LP than the semi-continuous culture in the same culture time in a 5 m^2^ ORP. However, the semi-continuous culture was more favorable for CO_2_ emission reduction than the two-stage induction culture. The semi-continuous culture prolonged culture period to reduce the supply of the original species.

#### Scaled up semi-continuous cultivation in 200 m^2^ ORP

**Fig. 4:**
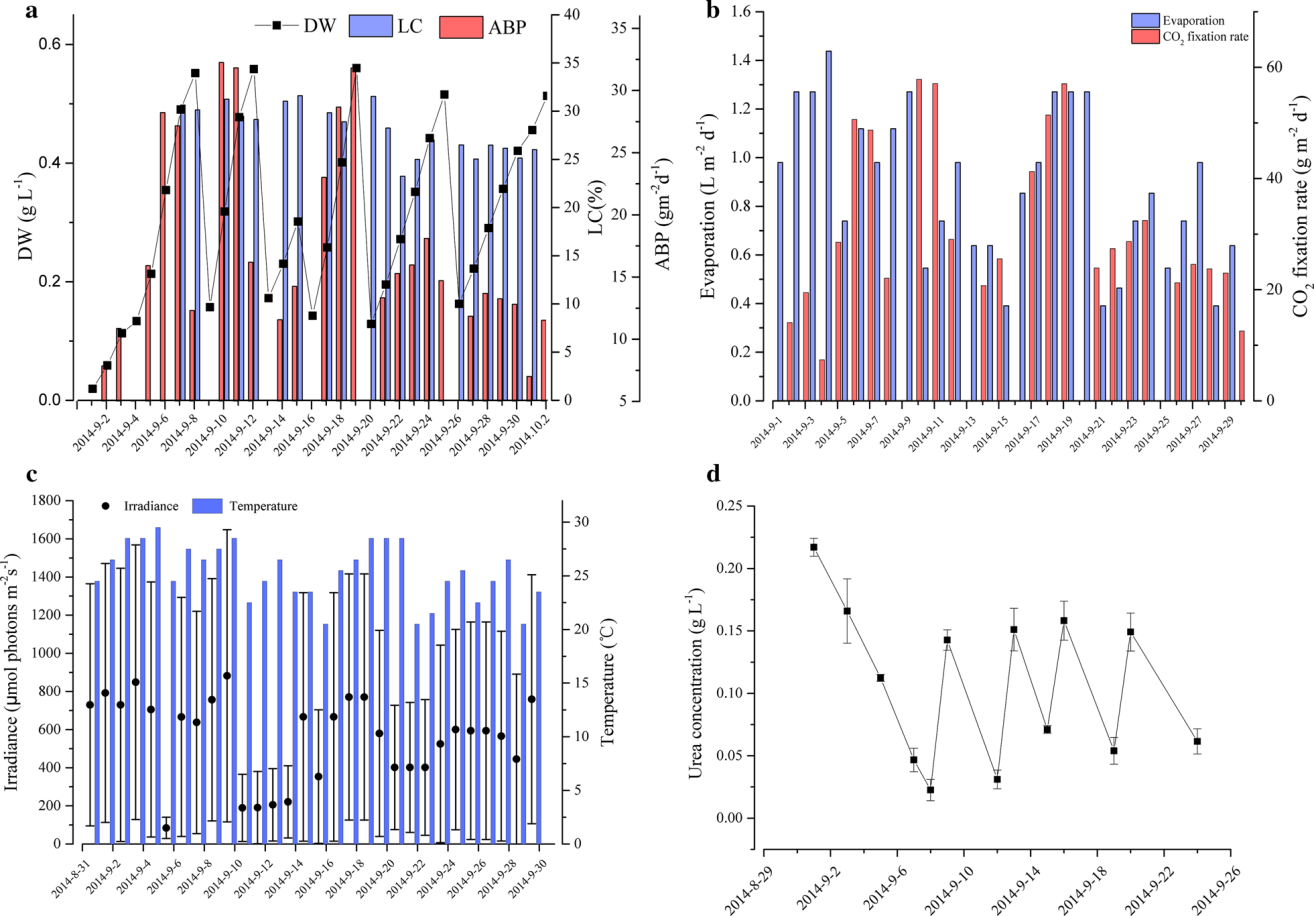
Time course profiles of dry weight, biomass productivity, lipid content (**a**), CO_2_ fixation rate, evaporation (**b**), water temperature, light intensity (**c**), and urea concentration (**d**) of *M. dybowskii* LB50 grown under semi-continuous mode in the 200 m^2^ ORP

Figure 4 shows the semi-continuous culture of *M. dybowskii* LB50 in a 200 m^2^ ORP (40,000 L) for a month. BP was 15.2 g m^−2^ day^−1^ during the initial growth (0–7 days). The highest BP was 26.8 g m^−2^ day^−1^ during the first cycle of semi-continuous culture, but was decreased at the second cycle, because of the rainy days (11–12 days, Fig. 4c). The average biomass productivity was 17 g m^−2^ day^−1^ (0–26 days, Table 3) after 1 month of semi-continuous culture at five cycles of replacement. The LC did not significantly change during the four cycles, but significantly decreased at the fifth passage. Therefore, the LP also decreased during fifth passage. The change in CO_2_ fixation rate was the same as that during biomass production. The average CO_2_ fixation rate was 30.8 or 33.9 g m^−2^ day^−1^ at 0–26 or 0–20 days (Table 3).

**Table 3:** LC, VBP, ABP, VLP, ALP, and CO_2_ fixation rate of *M. dybowskii* LB50 grown under semi-continuous mode in 200 m^2^ ORP

	LC (%)	VBP (mg L^−1^ day^−1^)	ABP (g m^−2^ day^−1^)	VLP (mg L^−1^ day^−1^)	ALP (g m^−2^ day^−1^)	CO_2_ fixation rate (mg L^−1^ day^−1^)	CO_2_ fixation rate (g m^−2^ day^−1^)
0–7 days	30.38 ± 0.35	76.02 ± 46.42	15.21 ± 9.28	23.09 ± 1.62	4.62 ± 0.33	136.35 ± 83.08	27.27 ± 16.64
8–10 days	29.96 ± 1.31	133.79 ± 45.77	26.76 ± 9.15	40.08 ± 5.99	8.01 ± 1.19	239.93 ± 82.08	47.98 ± 16.41
11–12 days	31.33 ± 0.41	64.54 ± 9.49	12.91 ± 1.89	20.22 ± 0.37	4.04 ± 0.08	115.73 ± 17.03	23.14 ± 3.41
13–15 days	29.37 ± 0.66	139.17 ± 22.27	27.83 ± 4.45	40.87 ± 1.49	8.17 ± 0.29	249.57 ± 39.95	49.92 ± 7.99
16–20 days	27.01 ± 3.66	77.40 ± 8.80	15.48 ± 1.97	20.91 ± 3.61	4.18 ± 0.72	138.81 ± 15.79	27.76 ± 3.15
21–26 days	25.58 ± 0.62	58.44 ± 12.17	11.69 ± 2.43	14.95 ± 0.75	1.99 ± 0.15	104.81 ± 21.82	20.96 ± 4.36
0–20 days	29.61 ± 1.62	94.41 ± 4.37	18.88 ± 2.73	27.95 ± 0.07	5.59 ± 0.04	169.29 ± 78.33	33.86 ± 15.67
0–26 days	28.44 ± 1.27	85.77 ± 17.49	17.15 ± 3.49	24.39 ± 2.12	4.88 ± 0.42	153.82 ± 31.31	30.76 ± 6.29

Evaporation occurred at 0.88 ± 0.31 L m^−2^ day^−1^ in the 200 m^2^ ORP, and the maximal evaporation rate was 1.44 L m^−2^ day^−1^ under high light intensity (128–1568 μmol m^−2^ s^−1^). Even during a rainy day, minimal evaporation loss of 0.39 m^−2^ day^−1^, which included the leakages and washout of the ORP, was found. Therefore, the average daily evaporation loss rate was 0.44%, and evaporation loss rate was 8.8–11.44% during the whole semi-continuous culture. Figure 4d shows that a small amount of urea can accumulate after each cycle of replacement. The accumulation of urea in the medium reached 0.05 g L^−1^ until the fourth cycle of semi-continuous culture. These results suggested that the growth and lipid of cells were affected by the accumulation of partial nutrients and the remaining death cells in the media as cycle times increased. Therefore, five cycles of repeated culture were conducted in this study. However, further scalable work can be continued for long-term cultivation with additional repeated times, considering the good performance observed in *M. dybowskii* LB50.

**Fig. 5:**
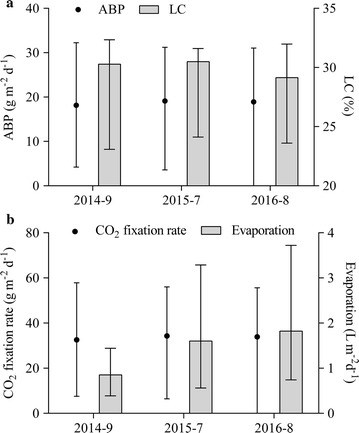
Biomass productivity and lipid content (**a**), CO_2_ fixation rate and evaporation (**b**) of *M. dybowskii* LB50 under 200 m^2^ ORP in 3 years

Three semi-continuous cultures of *M. dybowskii* LB50 in a 200 m^2^ ORP were performed thrice in September 2014, July 2015, and August 2016 (Fig. 5). *M. dybowskii* LB50 could exhibit stable growth for a month with semi-continuous culture. The biomass and LC were maintained at 18–20 g m^−2^ day^−1^ and 30%, respectively. The CO_2_ fixation rate remained at 33 g m^−2^ day^−1^, but the evaporation exhibited increased difference in various months. The evaporation rates were 0.39–1.44 L m^−2^ day^−1^ ($\bar{x}$ = 0.9 L m^−2^ day^−1^), 0.56–3.29 L m^−2^ day^−1^ ($\bar{x}$ = 1.6 L m^−2^ day^−1^), and 0.74–3.72 L m^−2^ day^−1^ ($\bar{x}$ = 1.8 L m^−2^ day^−1^) in September 2014, July 2015, and August 2016. The evaporation loss rate of a semi-continuous culture is 6.5–13%. Water resources are a potential limitation for microalgal culture, but evaporation affects its scale and sustainability [32]. Furthermore, regions with high BP receive high solar irradiance and thus result in high evaporation rates [33]. Evaporation of the ponds was assumed to occur at a rate of 0.4 cm day^−1^ (0.4 L m^−2^ day^−1^) [34]. In this case, further work on the water cyclic utilization and evaporation reduction can be conducted for sustainable cultivation because of the increased evaporation.

Replacement ratio or dilution ratio, the volume ratio of new medium to total culture, is an important parameter in semi-continuous culture because it influences microalgae growth and cell the biochemical components. Ho et al. [35] reported that BP increases with replacement ratio, but lipid causes the opposite effect. The 90% replacement group exhibited the highest overall LP among five replacement ratios (10, 30, 50, 70, and 90%). Some studies reported that a semi-batch process with a 50% medium replacement ratio is suitable for microalgal biomass production and CO_2_ fixation [13, 36]. In the current study, the LC was unaffected by the 2/3 replacement ratio mainly because of the high and long duration of light in the desert. Although the microalgal concentration in the reactor was not high, cells could grow rapidly.

Cycle time is another parameter affecting the continuity of semi-continuous culture. Previously, five to six cycles of repeated semi-continuous culture were conducted and resulted in inhibited growth or decreased LC [35, 37]. The LC of *Desmodesmus* sp. F2 significantly decreased at the sixth repeated cycle when five replacement ratios were adopted for semi-continuous cultivation for six repeated cycles [35]. In the 2/3 replacement test, the LC remained high throughout the five-cycle repeated course in 200 m^2^ ORPs.

Table 4 shows the LP of microalgae in large-scale culture outdoors. The largest scale was implemented in the cultivation of *N. salina* in the USA, and LP was 10.7 m^3^ ha^−1^ year^−1^ [38], followed by the cultivation of *M. dybowskii* LB50, *Graesiella* sp. WBG-1, and *M. dybowskii* Y2 in 200 m^2^ ORPs (40,000 L). The LPs (5.3 g m^−2^ day^−1^) of *M. dybowskii* LB50 and *M. dybowskii* Y2 were higher than those of *Graesiella* sp. WBG-1 (2.9 g m^−2^ day^−1^) and the others in ORPs and tubular photobioreactors. Increased CO_2_ fixation ability (CO_2_ fixation rate of 34 g m^−2^ day^−1^) was obtained under semi-continuous modes with ORPs in the desert area (Table 4). These results indicated that high biomass production was obtained and CO_2_ mitigation was feasible by microalgal culture in the desert. The volumetric LP (VLP) in ORPs was lower than that in photobioreactors (Table 4). Finally, all types of bioreactors must focus on the ALP in microalgae industry applications. In brief, the semi-continuous mode in ORPs is more practical than other operation modes in other bioreactors for long-term cultivation. Thus, it is suitable for oleaginous microalgae industry applications because it is economic, convenient, and demonstrates high ALP.

**Table 4:** Comparisons of biomass and lipid productivity of different sizes in some microalgae outdoors (culture volume > 100 L)

Strains	LC (%)	VBP (mg L^−1^ day^−1^)	ABP (g m^−2^ day^−1^)	VLP (mg L^−1^ day^−1^)	ALP (g m^−2^ day^−1^)	CO_2_ fixation rate		Bioreactor volume (L)	Culture	Location	References
						(mg L^−1^ day^−1^)	(g m^−2^ day^−1^)				
*N. gaditana*	18.6	590	15.4	110	2.9	1109^a^	28.9	TPs (340)	Dilution rate	Spain	[14]
	17.1	460	12.1	78.7	2.1	864.8^a^	22.8		Nutrient		
*Nannochloropsis* sp.	43	256		110		481.3^a^		GWP (590)	Nitrogen	Italy	[41]
*Chlorella* sp.	34.8	238		83		447^a^		BPs (120)	Batch	Australia	[11]
*T. suecica*	32	51.5		14.8		97^a^			CO_2_		[28]
*S. obliquus*	13.4	135	11.3	19	1.6	253.8^a^	21.3	HTP (500)	Semi-continuous	UK	[42]
*Nannochloropsis* sp.	28	16.75		4.69		31.50^a^		RCS (8000)	Flue gas	Shandong, China	[43]
*C*. sp. FC2 IITG	35.12	44.00	9.70	10.70	3.80	82.70^a^	18.20	OPs (300, 1.4 m^2^)	CO_2_	India	[15]
*S. rubescens*	13.8		4		0.6		7.6	RCS (9000)	Normal	USA	[44]
*N. salina*	16.33	204.2	24.5	33.3	4	383.9^a^	46.1	RCS (300)	CO_2_	Israel	[45]
*N. salina*	34.7	160		55.5		300.8^a^		AGSp (174,000)	Normol	USA	[46]
*B. braunii* TN101		169	33.8		8.2-13.0	317.7^a^	63.5	RCS (5000, 25 m^2^)	Semi-continuous	Malaysia	[10]
*B. braunii*	24	100		24^c^		188^a^		RCS (80)	Batch	India	[47]
*Graesiella* sp.	31.8	43.5	8.7	14.5	2.9	81.8^a^	16.4	RCS (40,000, 200 m^2^)	Batch (CO_2_)	Yunnan, China	[5]
*N. gaditana*	25.6	190	10.3–22.4	30.4	2.6–5.7	357.2^a^	19.4	RCS (792, 7.2 m^2^)	Continuous	Spain	[48]
*S. acutus* LB0414	21.5	42.9	3.5	9.2	0.8	80.7^a^	6.6	RCS (2278, 10 m^2^)	Batch	USA	[49]
*Tetraselmis* sp.	34.9	243	48.6	85	17	456.8^a^	31.9	RCS (200, 1 m^2^)	Batch	Australia	[50]
*S. obliquus* CNW-N (summer)		205.1		83.9^d^		358.8^b^		Tubular (60)	Batch	Taiwan, China	[51]
*S. obliquus* CNW-N (winter)		119.2		47.3^d^		208.7^b^		Tubular (60)	Batch	Taiwan, China	
*M. dybowskii* Y2	29.9	89.5	17.9	26.7	5.3	148.1^b^	29.6	RCS (40,000, 200 m^2^)	Semi-continuous	Inner Mongolia, China	[13]
*M. dybowskii* LB50	38.6	81.4	21.7	32.5	8.6	153.1^b^	40.8	Plastic bag (140 L)	NaCl-induced	Beijing, China	[16]
	30.34	50.1	10	15.2	3	89.9^b^	18.0	RCS	Batch	Inner Mongolia, China	This study
	30	90.6	18.1	27.2	5.4	162.5^b^	32.5	RCS	Semi-continuous		

*TPs* tubular photobioreactors, *GWP* green wall panel, *BPs* bag photobioreactors, *HTP* horizontal tubular photobioreactor, *TLP* thin-layer photobioreactor, *RCS* raceway cultivation system, *AGSp* algae growth system photobioreactor, *OPs* open ponds

^a^Calculated from the following equation: CO_2_ fixation rate = biomass productivity (mg L^−1^ day^−1^) × 1.88

^b^Calculated from the following equation: CO_2_ fixation rate = biomass productivity (mg L^−1^ day^−1^) × *C* (%) × 44/12

^c^For hydrocarbon

^d^For carbohydrate

### Energy consumption evaluation of outdoor cultivation in different culture modes

The biodiesel production from microalgae involved a course of cultivation, centrifugation, drying, and extraction via a conventional method. We assumed that 100,000 kg dry weight of biomass was produced within the year (270 days). Other parameters were included in our assessment according to the actual operation.

Table 5 shows that the net positive energy for oil production (1.34–2.72) and biomass production (1.41–2.52) in the two-stage salt induction or semi-continuous culture mode was higher than those in the batch mode in 5 m^2^ ORPs. Moreover, in the 200 m^2^ ORP, the net positive energy of oil production in the semi-continuous and batch modes was 1.52–2.69, indicating that the semi-continuous culture increased the biomass yield, but not the additional energy consumption. The NER of oil and biomass production increased with a scale-up of the culture system. In addition, the energy demand for producing 1 kg of biodiesel was 14.2–23.3 MJ under semi-continuous mode in 200 m^2^ ORP.

**Table 5:** Comparative energy analyses for biomass or bio-oil production based on 1 year of cultivating *M. dybowskii* LB50 via different culture modes under OPRs

Variable	5 m^2^			200 m^2^	
	Batch	Induction	Semi-continuous	Batch	Semi-continuous
Annual biomass production (kg year^−1^)	100,000	100,000	100,000	100,000	100,000
Volumetric productivity (g L^−1^ day^−1^) or (kg m^−3^day^−1^)^a^	0.04	0.04	0.07	0.08	0.09
Illuminated areal productivity (kg m^−2^ day^−1^)^a^	0.01	0.01	0.01	0.02	0.02
Reactor volume (m^3^)^b^	8413.68	8504.49	5513.10	4700.13	4087.97
Occupied area (m^2^)	42,087.54	42,522.43	27,557.32	23,500.66	20,439.87
Lipid content (%)^a^	30.85	39.39	30.84	30.13	30.13
Energy consumption for stirring (W m^−3^)^c^	3.72–12.5	3.72–12.5	3.72–12.5	3.72–12.5	3.72–12.5
Total energy for stirring (kWh months^−1^)^d^	7511.7–25,241.1	7592.8–25,513.4	4922.1–16,539.3	4196.2–14,100.3	3649.7–12,263.9
Total energy for biomass drying (kWh year^−1^)^e^	53,900.00	53,900.00	53,900.00	53,900.00	53,900.00
Total energy for oil recovery (kWh year^−1^)^f^	34,534.00	34,534.00	34,534.00	34,534.00	34,534.00
Total energy consumption for producing biomass	437.42–1011.85	440.05–1020.68	353.52–729.91	330–650.89	312.29–591.39
Total energy consumption for producing oil (GJ year^−1^)	561.74–1136.17	564.37–1145	477.84–854.24	454.32–775.22	436.61–715.71
Energy produced as oil (GJ year^−1^)^g^	1204.3	1537.8	1203.9	1176.3	1176.3
Energy produced as 100,000 kg biomass (GJ year^−1^)^h^	3155.33	3155.33	3155.33	3155.33	3155.33
NER for oil production^i^	2.14–1.06	2.72–1.34	2.52–1.41	2.59–1.52	2.69–1.64
NER for biomass production	7.21–3.12	7.17–3.09	8.93–4.32	9.56–4.85	10.1–5.34
Energy consumption for oil (MJ kg^−1^ bio-oil)	17.86–36.13	14.06–28.52	15.2–27.17	14.79–25.24	14.22–23.3
Energy consumption for oil (MJ MJ^−1^ bio-oil)	0.47–0.94	0.37–0.74	0.4–0.71	0.39–0.66	0.37–0.61

The assumed annual biomass production is 100,000 kg

^a^Data were based on this study

^b^Determined by dividing the illuminated area actual by production the volume of each unit

^c^3.72 W m^−3^ from Jorquera et al. [23]. 12.5 W m^−3^ from the actual date for the 200 m^2^ raceway pond

^d^Includes 8 h of daily pumping

^e^Stepan et al. [52]. 539 kWh ton^−1^ biomass

^f^Stephenson et al. [53]; Gao et al. [54]. 345.34 kWh ton^−1^ biomass

^g^Energy content of net oil yield (assumed value of 39.04 MJ kg^−1^); Jorquera et al. [23]

^h^Energy content of net biomass yield (assumed value of 31.55 MJ kg^−1^); Jorquera et al. [23]

^i^NER would be above 1 if including coproduct allocation [55]

**Fig. 6:**
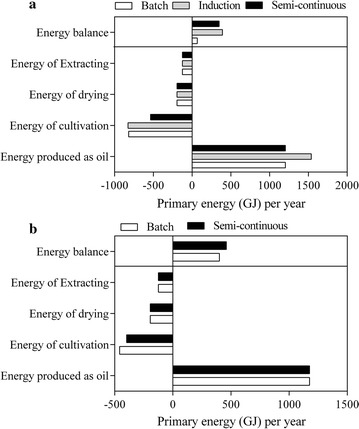
Comparative energy analyses for bio-oil production based on 1 year of *M. dybowskii* LB50 cultivation via different culture modes in 5 m^2^ ORPs (**a**), and 200 m^2^ ORPs (**b**). The assumed annual biomass production is 100,000 kg

Figure 6 shows that the energy consumption of cultivation assumed the highest proportion (55–72%) under any culture mode. The energy balance in the two-stage salt induction culture mode was higher than that in the other methods mainly due to the increase of LC by industrial salt induction to increase the energy produced by oil. The energy produced by oil was 1.27 times larger than that under other modes within the same biomass production (100,000 kg), but the energy balance was only about 10% higher than that under semi-continuous mode. These results demonstrate that the energy consumption of the cultivation process was increased and was reduced by scaling up. The energy balance thus increased after scaling up. Moreover, the energy balance under semi-continuous mode was five times higher than that under batch mode in 5 m^2^ ORPs and was 1.15 times higher in 200 m^2^ ORP. Therefore, reducing energy consumption by intermittent agitation or by optimizing mixing, mixing velocity, and paddlewheel must be prioritized to reduce the energy consumption of the entire industrial chain [39].

NER is associated with the type of culture system, and the NER of oil is generally less than 1 in tubular photobioreactors and greater than 1 in ORPs [23]. Ponnusamy et al. [40] reported that the energy demand for producing 1 kg of biodiesel is 28.23 MJ. Only 14–23 MJ was required for 1 kg of biodiesel in this study, which significantly decreased the energy consumption. He et al. [13] reported that the semi-continuous mode reduces the total costs (14.18 and 13.31$ gal^−1^) by 14.27 and 36.62% compared with the costs of batch mode in *M. dybowskii* Y2 and *Chlorella* sp. L1 in the desert area. Therefore, using semi-continuous culture mode with ORPs in the desert area can result in higher biomass, lower energy consumption, and lower costs compared with other culture modes.

## Conclusion

Three microalgae were investigated for their environmental tolerances and lipid production potential in ORP outdoors, and *M. dybowskii* LB50 can be efficiently cultivated using resources in the desert. Lipid production can be improved by using two-stage salt induction and semi-continuous culture modes in ORPs. After 3 years of operation, *M. dybowskii* LB50 was successfully and stably cultivated under semi-continuous mode for a month (five cycles of repeated culture) in 200 m^2^ ORPs in the desert, reducing the supply of the original species. The BP and CO_2_ fixation rates were maintained at 18 and 33 g m^−2^ day^−1^, respectively. The LC decreased only during the fifth cycle of repeated culture. Evaporation occurred at 0.9–1.8 L m^−2^ day^−1^ (6.5–13% of evaporation loss rate). Finally, using the semi-continuous and two-stage salt induction modes for cultivating *M. dybowskii*, LB50 can reduce energy consumption and increase energy balance via energy analysis of life cycle. Therefore, *M. dybowskii* LB50 is a promising candidate for the large-scale, outdoor production of biodiesel feedstock in desert areas. The outdoor ORP cultivation system together with the semi-continuous culture method in desert areas is a suitable strategy to further decrease the cultivation cost and increase the biomass/oil production and CO_2_ emission potential of *M. dybowskii* LB50.

## Additional files

**Additional file 1: Figure S1.** Outdoor cultivation system of large-scale raceway ponds.

**Additional file 2: Table S1.** Ingredients of industrial salt.

**Additional file 3: Table S2.** Elemental analysis of *M. dybowskii* LB50, *Micractinium* sp. XJ-2, and *P. falcata* XJ-176.

**Additional file 4: Table S3.** LC, BP, and LP of *M. dybowskii* LB50, *Micractinium* sp. XJ-2 and *P. falcata* XJ-176 cultivated indoors.

**Additional file 5: Table S4.** LC, VBP, ABP, VLP, ALP, and CO_2_ fixation rate of *M. dybowskii* LB50, *Micractinium* sp. XJ-2, and *P. falcata* XJ-176 grown in 5 m^2^ ORPs.

**Additional file 6: Figure S2.** Irradiance, temperature and pH of three microalgae in 5 m^2^ ORPs.

## Abbreviations

ORP: open raceway pond
BP: biomass productivity
DW: dry weight
LC: lipid content
LP: lipid productivity
VBP: volumetric biomass productivity
ABP: areal biomass productivity
VLP: volumetric lipid productivity
ALP: areal lipid productivity
NER: net energy ratio
OD: optical density
