# Supplementary material for: Feasibility of biodiesel production and CO2 emission reduction by Monoraphidium dybowskii LB50 under semi-continuous culture with open raceway ponds in the desert area
Source: Biotechnol Biofuels. 2018 Apr 2;11:82. doi: 10.1186/s13068-018-1068-1 (PMC5879568; doi:10.1186/s13068-018-1068-1)
Supplement: Supplementary file 1 — Additional file 1: Figure S1. Outdoor cultivation system of large-scale raceway ponds. [file 13068_2018_1068_MOESM1_ESM.docx]

## Additional file 1: Figure S1. Outdoor cultivation system of large-scale raceway ponds


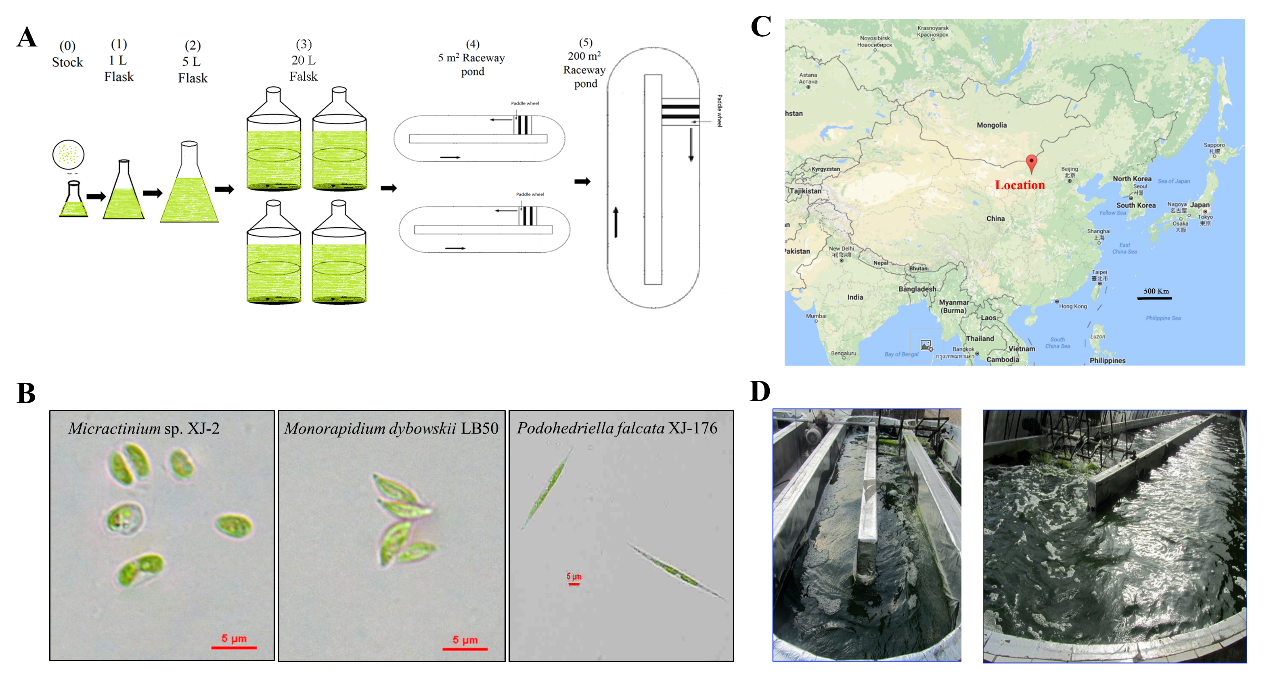


Fig. S1 Outdoor cultivation system of large-scale raceway ponds: Scale-up steps, (A); Strans, (B); cultivation location, (C); and 5 and 200 m^2^ ORPs (D).
