# Supplementary material for: Feasibility of biodiesel production and CO2 emission reduction by Monoraphidium dybowskii LB50 under semi-continuous culture with open raceway ponds in the desert area
Source: Biotechnol Biofuels. 2018 Apr 2;11:82. doi: 10.1186/s13068-018-1068-1 (PMC5879568; doi:10.1186/s13068-018-1068-1)
Supplement: Supplementary file 2 — Additional file 2: Table S1. Ingredients of industrial salt. [file 13068_2018_1068_MOESM2_ESM.docx]

## Additional file 2: Table S1. Ingredients of industrial salt

Table S1 Ingredients of industrial salt

|  | NaCl (%) | Water (%) | Insoluble residue (%) | Ca^2+^ and Mg^2+^ (%) | SO_4_^2-^ (%) |
| --- | --- | --- | --- | --- | --- |
| Industrial salt | 97.50 | 0.80 | 0.20 | 0.60 | 0.90 |
