# Supplementary material for: Feasibility of biodiesel production and CO2 emission reduction by Monoraphidium dybowskii LB50 under semi-continuous culture with open raceway ponds in the desert area
Source: Biotechnol Biofuels. 2018 Apr 2;11:82. doi: 10.1186/s13068-018-1068-1 (PMC5879568; doi:10.1186/s13068-018-1068-1)
Supplement: Supplementary file 3 — Additional file 3: Table S2. Elemental analysis of M. dybowskii LB50, Micractinium sp. XJ-2, and P. falcata XJ-176. [file 13068_2018_1068_MOESM3_ESM.docx]

## Additional file 3: Table S2. Elemental analysis of three microalgae indoors.

**Table S2** Elemental analysis of *M. dybowskii* LB50, *Micractinium* sp. XJ-2, and *P. falcata* XJ-176.

|  | *M.dybowskii* LB50 | *Micractinium* sp. XJ-2 | *P. falcata* XJ-176 |
| --- | --- | --- | --- |
| C (%) | 48.91 | 47.25 | 51.37 |
| H (%) | 7.24 | 6.61 | 6.38 |
| O (%) | 28.04 | 28.12 | 27.53 |
| N (%) | 7.63 | 8.31 | 7.53 |
