# Supplementary material for: Feasibility of biodiesel production and CO2 emission reduction by Monoraphidium dybowskii LB50 under semi-continuous culture with open raceway ponds in the desert area
Source: Biotechnol Biofuels. 2018 Apr 2;11:82. doi: 10.1186/s13068-018-1068-1 (PMC5879568; doi:10.1186/s13068-018-1068-1)
Supplement: Supplementary file 4 — Additional file 4: Table S3. LC, BP, and LP of M. dybowskii LB50, Micractinium sp. XJ-2 and P. falcata XJ-176 cultivated indoors. [file 13068_2018_1068_MOESM4_ESM.docx]

## Additional file 4: Table S3. LC, BP, and LP of three microalgae cultivated indoors.

**Table S3** LC, BP, and LP of *M. dybowskii* LB50, *Micractinium* sp. XJ-2, and *P. falcata* XJ-176 cultivated indoors.

| Strains | LC (%) | BP (mg L^-1^ d^-1^) | LP (mg L^-1^ d^-1^) |
| --- | --- | --- | --- |
| *M. dybowskii* LB50 | 33.97±1.73 | 92.01±5.22 | 31.25±1.75 |
| *Micractinium* sp. XJ-2 | 30.91±0.28 | 106.59±4.91 | 32.95±1.52 |
| *P. falcata* XJ-176 | 32.88±0.37 | 112.56±5.18 | 37.01±1.92 |
