# Supplementary material for: Feasibility of biodiesel production and CO2 emission reduction by Monoraphidium dybowskii LB50 under semi-continuous culture with open raceway ponds in the desert area
Source: Biotechnol Biofuels. 2018 Apr 2;11:82. doi: 10.1186/s13068-018-1068-1 (PMC5879568; doi:10.1186/s13068-018-1068-1)
Supplement: Supplementary file 5 — Additional file 5: Table S4. LC, VBP, ABP, VLP, ALP, and CO2 fixation rate of M. dybowskii LB50, Micractinium sp. XJ-2, and P. falcata XJ-176 grown in 5 m2 ORPs. [file 13068_2018_1068_MOESM5_ESM.docx]

## Additional file 5: Table S4. LC, VBP, ABP, VLP, ALP and CO_2_ fixation rate of three microalgae grown in 5 m^2^ ORPs.

**Table S4** LC, VBP, ABP, VLP, ALP and CO_2_ fixation rate of *M. dybowskii* LB50, *Micractinium* sp. XJ-2, and *P. falcata* XJ-176 grown in 5 m^2^ ORPs.

|  | *M. dybowskii* LB50 | *Micractinium* sp. XJ-2 | *P. falcata* XJ-176 |
| --- | --- | --- | --- |
| LC (%) | 31.04±0.99 | 27.05±0.67 | 32.05±0.72 |
| VBP (mg L^-1^ d^-1^) | 41.99±1.64 | 43.05±1.30 | 10.19±1.27 |
| ABP (g m^-2^d^-1^) | 8.39±0.86 | 8.60±0.56 | 2.04±0.28 |
| VLP (mg L^-1^d^-1^) | 13.03±0.42 | 11.40±0.32 | 3.27±0.07 |
| ALP (g m^-2^d^-1^) | 2.6±0.08 | 2.28±0.06 | 0.66±0.01 |
| CO_2_ fixation rate (mg L^-1^ d^-1^) | 59.17±8.27 | 40.66±0.07 | 19.19±5.25 |
| CO_2_ fixation rate (g m^-2^ d^-1^) | 11.83±1.65 | 8.13±0.01 | 3.84±1.05 |
