# Supplementary material for: Feasibility of biodiesel production and CO2 emission reduction by Monoraphidium dybowskii LB50 under semi-continuous culture with open raceway ponds in the desert area
Source: Biotechnol Biofuels. 2018 Apr 2;11:82. doi: 10.1186/s13068-018-1068-1 (PMC5879568; doi:10.1186/s13068-018-1068-1)
Supplement: Supplementary file 6 — Additional file 6: Figure S2. Irradiance, temperature and pH of three microalgae in 5 m2 ORPs. [file 13068_2018_1068_MOESM6_ESM.docx]

## Additional file 6: Figure S2. Irradiance, temperature, and pH of three microalgae in 5 m^2^ ORPs.

Fig. S2 Irradiance, temperature, and pH of *M. dybowskii* LB50 (A, B), *Micractinium* sp. XJ-2 (C, D), and *P. falcata* XJ-176 (E, F) in 5 m^2^ ORPs.
